# Shade matters: heat stress alleviation in Gyr and Girolando cows through silvopastoral management in tropical conditions

**DOI:** 10.1007/s00484-025-03063-7

**Published:** 2026-01-14

**Authors:** Concepta McManus, Felipe Pimentel, Vinícius Silva Junqueira, Luiz Carlos Balbino, Luiz Adriano Maia Cordeiro, Francisco Bernal, Vanessa Peripolli, Isabel Cristina Ferreira

**Affiliations:** 1 Universidade de Brasília, Campus Darcy Ribeiro, Brasília, Distrito Federal 70910-900 Brasil; 2 Centro de Energia Nuclear na Agricultura (CENA), Universidade de São Paulo, Piracicaba, São Paulo, 13416-000 Brasil; 3 Universidade Anhembi Morumbi, Campus Avenida Paulista, São Paulo, 01310-200 Brasil; 4 Bayer Crop Science, Uberlândia, 38057-049 Minas Gerais Brasil; 5 Embrapa Cerrados, Planaltina, Distrito Federal 73310-970 Brasil; 6 Embrapa Sede, Parque Estação Biológica, Brasília, Distrito Federal 70770- 901 Brasil; 7 Instituto Federal Catarinense, Campus Araquari, Araquari, Santa Catarina 89245-000 Brasil; 8 Embrapa Cerrados, Uberaba, Minas Gerais 38001-970 Brasil

**Keywords:** Animal welfare, Heat stress, Physiology, Silvopastoral, Sustainable agriculture, Thermography

## Abstract

Silvopastoral systems integrating tree cover into pasturelands offer promising strategies to mitigate heat stress in tropical livestock production. This study evaluated the effects of shade from *Eucalyptus urograndis* on thermal comfort indices in Gyr and Girolando dairy cattle in the Brazilian Cerrado. Forty-eight lactating cows (24 Gir, 24 Girolando) were monitored over two years in two contrasting environments: no shade (full sun) and shade (silvopasture). Thermal indices, including rectal and surface temperatures (measured via infrared thermography), respiration rates, and panting scores, were analyzed alongside environmental data, such as the Temperature-Humidity Index (THI). Cattle under shade consistently exhibited lower body temperatures, respiration rates, and panting scores, with significant differences amplified under heat stress conditions (THI > 74). Though both benefited from shade, Gyr cattle demonstrated better thermoregulatory responses than Girolando. Discriminant analysis accurately classified animals by system (> 92%) and breed (~ 71%) based on physiological traits. These results confirm that tree shade significantly enhances animal comfort in tropical pasture systems and supports the implementation of silvopastoral practices to improve dairy cattle welfare and resilience under climate stress.

**Supplementary Information:**

The online version contains supplementary material available at 10.1007/s00484-025-03063-7.

## Introduction

Integrated forest-livestock systems (i.e., silvopastoral systems) combine trees, pasture, and livestock to mimic natural ecosystems and promote sustainable agriculture (Leite-Moraes et al. 2023). These systems offer numerous benefits, particularly in enhancing animal comfort and welfare, and play a crucial role in carbon sequestration within the production system (Leite et al. 2023; Rivera et al. 2024). Trees provide shade, supporting thermoregulation, thereby reducing the incidence of heat stress (Cândido et al. 2023). Consequently, this improvement enhances feed intake, increases productive performance, and contributes to the overall health and well-being of the herd (Lemes et al. 2021; Rivera et al. 2024). Additionally, the presence of trees helps protect livestock from wind, rain, and cold, offering year-round shelter (Richards et al. 2024). Some tree species used in these systems also provide high-protein forage, improving the nutritional quality of the diet of the animals, and may contain natural compounds that reduce internal parasites (Vandermeulen et al. 2018).

The environments created by forest-livestock systems tend to be more diverse than those solely based on pasture (Mauricio et al. 2019), which reduces parasite hotspots and promotes natural behaviours in animals (Améndola et al. 2016). Beyond animal comfort, integrated systems contribute to environmental sustainability by sequestering carbon, improving soil fertility, enhancing biodiversity, and increasing land productivity (Peri et al. 2017). These systems, commonly practiced in Latin America, Africa, and Southeast Asia, demonstrate how integrating forestry and livestock can lead to more resilient, productive, and welfare-friendly agricultural practices.

The adaptability of the animals to the environment and the animal heat tolerance are determined by physiological parameters, such as respiration rate and body temperature (Costa et al. 2015; McManus et al. 2023). Nevertheless, measuring these variables can cause stress itself. Alternative phenotyping methods include the use of thermographic imaging, which can indicate circulatory changes induced by increased body temperature related to environmental heat stress, resulting in changes in the surface temperatures of the animals (McManus et al. 2016). The primary benefits of this tool are to enhance animal welfare during evaluation and to perform a larger number of assessments in a shorter time, without requiring animal restraint (Daltro et al. 2017; Vieira et al. 2023a, b). This study, therefore, aimed to assess the physiological, hormonal, and thermoregulatory responses under continuous grazing within silvopastoral and traditional systems, with the goal of identifying indicators of heat stress tolerance in tropical environments.

## Materials and methods

### Experiment description

The experiment was conducted at Embrapa Cerrados (Center of Technology for Dairy Zebu Breeds-CTZL), Brasília, DF, Brazil (15°57’09” S, 48°08’12” W), located in the central region of the country within the Cerrado biome over 12 months.

To assess the influence of the silvopastoral system on animal and environmental parameters, a randomized block design was implemented with two treatments over a total area of 16 hectares. The first treatment served as a control and covered half of the area, 8 hectares, where cows grazed under full sun conditions. The second treatment (8 ha) featured a silvopastoral system integrating pasture and forest, where cows grazed under the shade of *Eucalyptus urograndis* trees. The shaded and no shade treatments were located in contiguous areas within the same experimental site, under similar soil and grazing conditions. In both treatments, the pasture consisted of *Panicum maximum* cv. Mombaça. Forage allowance was maintained at approximately 40–50 kg DM/cow/day (8–10% of body weight) in both shaded and no shade paddocks, ensuring similar pasture offer between systems. Additionally, non-experimental animals were added as control animals to maintain sward height when needed. Henceforth, to streamline and standardise terminology within the paper, the term “shade” will refer to the silvopastoral system, whereas “no shade” will denote the traditional system exposed to full sunlight.

In the shaded system, eucalyptus trees (~ 6 years old and ~ 20 m tall) were planted in single rows with 1.5 m spacing between trees and 25 m between rows, oriented roughly east-west. This arrangement resulted in a density of 267 trees per hectare, providing approximately 8% canopy cover. The layout adhered to Embrapa’s technical recommendations (Paciullo et al. 2021), ensuring sufficient space for livestock, crops, and machinery.

To evaluate the impact on livestock, a total of 48 lactating cows aged between 3 and 6 years of age − 24 Gyr and 24 Girolando - were monitored over two years under continuous grazing conditions. Each cow was considered an experimental unit, and phenotypic data were collected individually throughout the experiment. Half the animals from each breed were randomly assigned to one of two management systems - shade or no shade - ensuring that both genetic groups were equally represented in each condition. Cows were walked approximately 500 m from the pasture to a herringbone milking parlour and milked twice daily, at 6 am and 5 pm. Individual milk yield was measured fortnightly using an electronic measure installed directly on the milking machine. Data on the cows also included their pregnancy status at the time of measurement (pregnant or not) and the number of days in milk (DIM).

Hourly climate data, including air temperature, humidity, wet and dry bulb temperatures, rainfall, ultraviolet index, and wind speed, were obtained from NASA Power database (https://power.larc.nasa.gov/data-access-viewer/) and averaged for the morning – after milking (7 am to 10 am) and afternoon – before milking (2 pm to 4 pm). This dataset provides grid estimates (0.5° × 0.5°, ~ 50 km resolution) referenced to the geographic coordinates of the experimental site.

### Skin and coat phenotypes

Skin and coat traits, including thickness, hair density, hair length, and colour, were assessed following the method described by Silva (2000). Skin (ST) and coat thickness (CT) were measured using an adipometer with 0.1 mm precision at multiple anatomical regions: scapular area, dorsum, flank, and hind leg. The hair density (HC) was determined by collecting samples (1 cm²) from the scapular region using forceps. Samples were stored in labelled envelopes for subsequent counting and measurement of the ten longest hairs. Hairs were spread on contrasting backgrounds (white paper for dark hairs, black for white hairs) for analysis. Hair length (HL) was calculated as the average of the ten longest hairs per sample, measured with a standard ruler. Skin and coat pigmentation were evaluated each year using the CIELCH system and a Minolta^®^ CR-10 colorimeter. This device measures L, which represents Lightness (ranging from 0 to 100), C for Chroma (indicating the intensity or purity of color), and H for Hue (the position on the color wheel, typically in degrees from 0 to 360°). LCH is a cylindrical transformation of CIELab color space, where L indicates lightness, C indicates chroma (C = $\:\sqrt{{a}^{2}+{b}^{2}}$); and H indicates hue angle (H = $\:arctan\frac{b}{a}$, where 𝑎 and 𝑏 are the chromaticity coordinates from CIELab).

The cows were also measured for wither height (WH), body length (BL), shin circumference (SC), and chest circumference (CC), using a measuring tape and a hipometer.

### Thermographic and respiration measurements

The animals were taken calmly to a covered area immediately to obtain physiological measurements, so no time was allowed for the animals to adapt to the shaded area before recordings were completed. This procedure was adopted because sunlight on the animals alters the conductivity and emissivity of the thermal image (Stewart et al. 2005; McManus et al. 2016). Thermographic images of the animals and bare ground were captured using a FLIR T300 infrared camera (emissivity: 0.98; temperature range: − 20 to 650 °C; accuracy: ±2%). Temperature data were analysed with QuickReport software for regions including the udder (Temp_Udder), rump (Temp_Rump), flank (Temp_Flank), eye (Temp_Eye), neck (Temp_Neck), and Muzzle (Temp_Muzzle). Two images per cow (lateral body and udder views) were taken at 7 am and 3 pm from a distance of 2 m. The site was dry with a concrete floor and a galvanised roof.

Rectal temperature (RT) was measured using a standard veterinary clinical thermometer. In contrast, coat temperature (TC) was recorded simultaneously using an infrared laser thermometer at the time the image was captured (Cruz Junior et al. 2015).

Respiration rate (RR) was assessed using a five-point scale ranging from 0 to 4, where 0 indicated normal breathing (≤ 30 breaths per min), 1 - slightly increased (31–40 breaths per min), 2 showed moderate panting and/or presence of small amount of drool or saliva (41–50 breaths per min), 3 had saliva usually present, panting hard with mouth open (51–60 breaths per min), and 4 showed severe panting with open mouth, protruding tongue, excessive drooling, and generally, extended neck (> 60 breaths per min), as described by Mader et al. (2006) and Dalcin et al. (2016). Scores were recorded individually for each cow during the morning and afternoon periods, under their respective grazing systems (no shade or shade).

### Thermal index

Environmental data were downloaded from NASA Power, which monitored environmental parameters such as air temperature, humidity, wet and dry bulb temperatures, rainfall, and wind speed. The Temperature-Humidity Index (THI) was calculated according to the formula proposed by the NRC (1971): $\:THI=\left(1.8\:\times\:DBT+32\right)-[\left(0.55-0.0055\times\:RH\right)\times\:(1.8\times\:DBT-26\left)\right]$, where DBT represents the dry bulb temperature (°C), and RH is the relative humidity (%). Based on the classification proposed by Hahn et al. (2009), THI values were interpreted as follows: normal (≤ 74), alert (75–78), danger (79–83), and emergency (> 84). For analysis, values were classified into two categories: heat stress (THI > 74) and no-stress (THI ≤ 74) conditions.

### Hormone measures

Thyroid hormone levels (T3 and T4) were measured monthly by ELISA assay (Foresight^®^ ACON Labs, San Diego) for in vitro quantitative determination of the total amount of binding sites available for the thyroid hormones, using serum collected by vein puncture before morning feeding from each animal. For each collection, approximately 10 mL of blood was collected into vacutainer tubes without anticoagulant (serum separator tubes) and allowed to clot at room temperature for about 30 min. Tubes were then centrifuged at 1,500 × g for 15 min, and the serum was aliquoted into cryovials and stored at − 20 °C until analysis.

### Statistical analysis

Six distinct statistical analyses were conducted to evaluate different aspects of the data collected in this experiment. First, an analysis of variance was performed using a mixed model approach to analyze the traits measured in this study. The model included fixed effects for breeds (Gyr and Girolando) and grazing systems (no shade and shade), as well as their interaction (breed × system) and whether the cow was primiparous or multiparous, and pregnancy status (0 or 1). Covariates such as milk yield, temperature-humidity index (THI), wind speed, and days in milk (DIM) were included as linear covariates. Cow was treated as a random effect to account for repeated measures on each animal. Second, a broken-line regression analysis was conducted to estimate the inflexion point at which physiological responses begin to change, with models fitted separately for each breed and grazing system. This approach allows for identifying threshold values in temperature or THI beyond which animals exhibit sharper stress responses.

Third, logistic regression was used to compare animals under non-stressful conditions and those exposed to thermal stress. In this study, a non-stressful condition was defined for THI < 74. Based on the methodology described by Schober and Vetter (2021), this analysis estimated the probability of stress-related outcomes based on key predictor variables and helps define the physiological thresholds associated with heat load.

Fourth, discriminant analysis was applied to classify animals into predefined groups based on their physiological and morphometric traits. This technique identifies the most relevant variables contributing to group differentiation, providing insight into breed- or grazing system-specific responses, as described by McManus et al. (2011).

Fifth, path analysis was employed to examine cause-and-effect relationships among the environmental, genetic groups and physiological variables influencing thermal stress. Following Tyasi et al. (2020), this multivariate method identified direct and indirect effects and clarified the underlying mechanisms involved in stress responses. Finally, a chi-squared test was applied to compare the distribution of animals with altered respiration rates between production systems and genetic groups, offering insight into the association between management practices and physiological outcomes. Data normality was confirmed using the Shapiro-Wilk test. All statistical analyses were performed using SAS v9.4 (SAS Institute, Cary, NC, USA). The figure generated from the path analysis results was created with the *DiagrammeR* R Package.

## Results

### Effects of grazing system and breed on rectal temperature, respiration rate, and surface temperatures

The grazing system (shade vs. no shade) significantly influenced rectal temperature (*p* < 0.001) and all surface temperature measurements (udder, rump, flank, neck, eye, and muzzle), but had no significant effect on respiration rate (Table 1). The breed had a significant effect on respiration rate, as well as on rectal temperature and most surface temperatures, particularly the rump, flank, neck, eye, and muzzle (Table 1). However, no significant interaction was observed between the grazing system and genetic group for any of the traits evaluated (Table 1).

**Table 1:** Statistical significance (based on F-statistics) from the analysis of variance evaluating the effects of grazing system (no shade vs. shade), breed (Gyr and Girolando), and environmental and physiological factors on rectal temperature, respiration rate, and surface temperatures at different body regions (udder, rump, flank, neck, eye, and muzzle)

Factor^1^	Rectal Temperature	Respiration Rate	Surface Temperature					
			Udder	Rump	Flank	Neck	Eye	Muzzle
Grazing system	***	Ns	***	***	***	***	***	***
Breed	*	***	Ns	***	***	*	***	***
Grazing system × breed	Ns	Ns	Ns	Ns	Ns	Ns	Ns	Ns
Gestation	Ns	Ns	Ns	Ns	Ns	Ns	Ns	Ns
Time of day	*	***	***	***	***	***	***	***
Days in milk	Ns	Ns	Ns	Ns	Ns	Ns	Ns	Ns
Number of gestations	Ns	Ns	Ns	Ns	Ns	Ns	Ns	Ns
Pregnancy	Ns	Ns	Ns	Ns	Ns	Ns	Ns	Ns
Milk production	Ns	Ns	Ns	Ns	Ns	Ns	Ns	Ns
THI	Ns	Ns	*	***	*	***	Ns	**
Wind speed	***	Ns	***	Ns	Ns	Ns	Ns	Ns
Ultraviolet index	*	***	***	Ns	*	Ns	Ns	Ns

*THI* temperature-humidity index. Gestation refers to primiparous and multiparous

F-statistics significance: *** *p* < 0.001; ** *p* < 0.01; * *p* < 0.05; *Ns* not significant

Among the physiological and environmental covariates, time of day had a significant effect on all variables, including rectal temperature, respiration rate, and surface temperatures (Table 1). The temperature-humidity index influenced several surface temperatures (udder, rump, flank, neck, and muzzle), though it did not significantly affect rectal temperature, respiration rate, and eye temperature. Wind speed was associated with changes in rectal temperature and udder temperature, while ultraviolet index significantly affected rectal temperature, respiration rate, udder temperature, and flank temperature.

No significant effects were found for physiological status indicators, including gestation status (i.e., primiparous and multiparous), number of gestations, pregnancy status, days in milk, or milk production, suggesting that these factors did not influence thermal or respiration responses under the conditions of this study (Table 1).

**Fig. 1:**
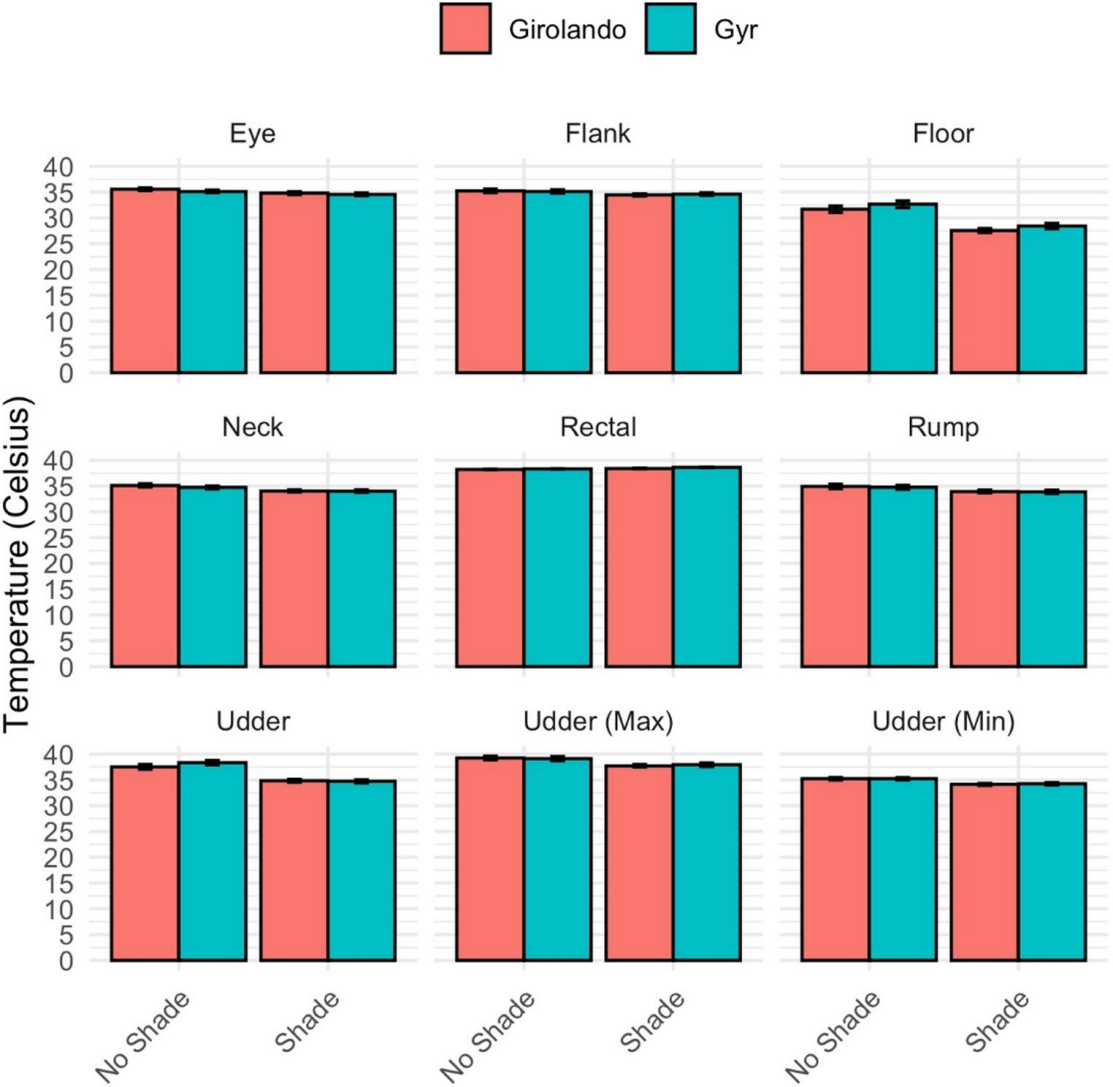
Least squares means of the rectal and surface temperature traits collected in an experiment evaluating the effects of grazing system (shade vs. no shade) in different breeds (Gyr and Girolando). Temperature variables are expressed in celsius. Bars represent ± standard error

Cows kept under shade exhibited higher rectal temperatures (Gyr: 38.17 °C; Girolando: 38.12 °C) compared to those under non-shaded (Gyr: 37.90 °C; Girolando: 37.76 °C), indicating that shaded environments, while reducing solar heat load, could also limit air movement and thus reduce heat dissipation (*p* < 0.001) (Fig. 1). However, respiration rate was slightly lower in Gyr cows across both shade and non-shade systems and consistently higher in Girolando cows, potentially indicating a greater sensitivity to heat stress in the latter (*p* < 0.05). Surface temperatures were generally lower under shade conditions. For example, the average udder surface temperature was approximately 2 °C lower in shaded animals (Gyr: 34.09 °C; Girolando: 34.44 °C) than in those under non-shaded (Gyr: 36.12 °C; Girolando: 35.95 °C; *p* < 0.001). Similar patterns were observed for the maximum and minimum udder temperatures and the rump, flank, neck, eye, and muzzle regions. Ground temperature was also markedly lower under shade (approximately 26 °C) than in non-shaded conditions (about 30 °C), likely contributing to reduced animal heat load.

**Fig. 2:**
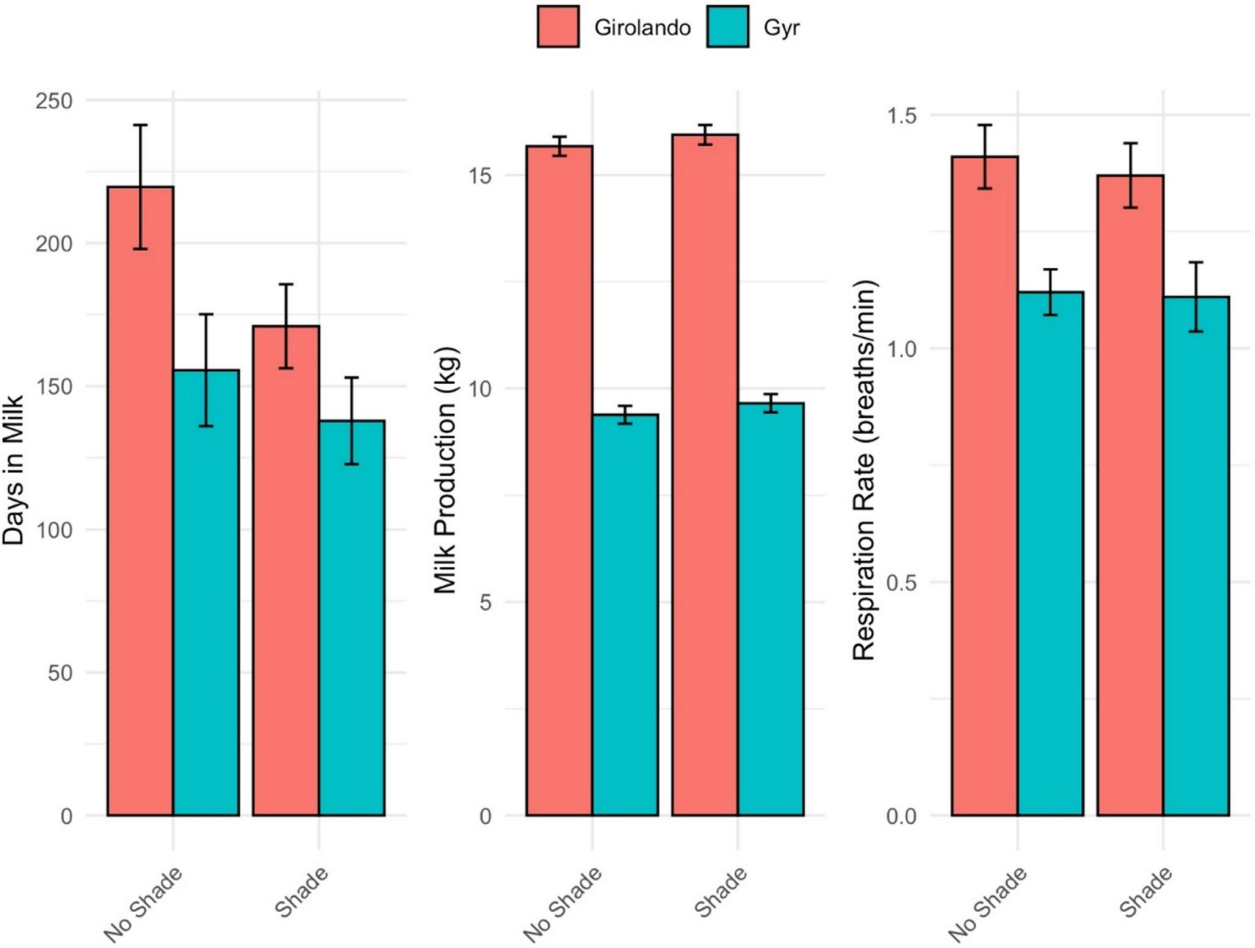
Least squares means of the production (days in milk and milk production) and respiration rate collected in an experiment evaluating the effects of grazing system (shade vs. no shade) in different breeds (Gyr and Girolando). Temperature variables are expressed in celsius. Bars represent ± standard error

Regarding physiological and productive traits, Girolando cows had more days in milk and greater milk production in both systems, particularly under shade (15.94 kg/day vs. 9.65 kg/day in Gyr cows; *p* < 0.001) (Fig. 2). The number of gestations was similar between groups, with a slight increase observed in Girolando cows under the no shade system. These results suggest that while Girolando cows are more productive, they display more pronounced physiological responses to heat stress, as expected. In contrast, shaded environments help mitigate internal and external body temperatures in both genetic groups.

The distribution of respiration rate classes revealed notable differences between breeds and grazing systems (Fig. 2). Girolando cows had more animals in the elevated respiration classes (classes 1, 2, and 3) than Gyr cows. Specifically, 27.53% of Girolando cows were in class 1, versus 23.70% for Gyr, and 11.10% of Girolando cows were in class 2, compared to 6.44% for Gyr. The difference was particularly pronounced in class 3, where 2.19% of Girolando cows exhibited the highest respiration rate category, while only 0.55% of Gyr cows fell into this group. These findings suggest that Girolando cows are more physiologically responsive to heat, as indicated by elevated respiration rates.

When comparing grazing systems, animals under shade tended to have slightly higher proportions in classes 1 and 3 (25.21% and 1.51%, respectively) than those under non-shaded conditions (26.03% and 1.23%, respectively). Conversely, the proportion of animals in the lowest respiration class (class 0) was higher in shaded conditions (15.07%) compared to non-shaded conditions (13.42%). These results suggest that shading may slightly reduce the proportion of cows in extreme respiration stress, but does not eliminate elevated physiological responses of lactating cows under heat.

### Responses to heat stress

The analysis of physiological and surface temperature traits between thermal stress categories (no stress vs. stress) showed clear differences between the breeds (Table 2). During stress conditions, both Gyr and Girolando cows exhibited elevated rectal temperatures, with Gyr cows increasing from 37.79 °C to 38.52 °C and Girolando from 37.78 °C to 38.29 °C. The respiration rate increased significantly under stress, especially in Girolando cows, rising from 0.88 to 1.44 (a 63% increase). In comparison, Gyr cows’ rate increased from 0.63 to 1.12 (a 77% increase), indicating both breeds exhibited a notable physiological response to heat; however, Girolando demonstrated a higher sensitivity to heat stress, as illustrated by their respiration rate levels. All surface temperatures (udder, rump, flank, neck, eye, and muzzle) rose under stress for both breeds. The udder temperature of Gyr cows increased from 33.97 °C to 37.17 °C, while for Girolando it went from 34.15 °C to 36.92 °C. Maximum udder temperatures rose by over 3 °C in both breeds, and similar increases were noted in minimum udder temperatures and other body areas. For instance, eye temperature climbed from 32.66 °C to 35.76 °C in Gyr cows and from 33.46 °C to 35.94 °C in Girolando cows. The ground temperature was higher (*p* < 0.001) during stress (32.41 °C for Gyr and 31.79 °C for Girolando; *p* > 0.05) compared to no-stress conditions (25.47 °C and 25.14 °C, respectively); *p* > 0.05, indicating an increase in environmental heat load. Milk production remained stable across stress categories despite the physiological differences, with no notable change in either breed. Days in milk and number of gestations also showed minimal variation between stress levels, indicating that these factors did not significantly influence the physiological responses observed. Overall, both breeds experienced measurable increases in internal and surface body temperatures and respiration rate under heat stress. Girolando cows consistently showed higher respiration responses, suggesting greater sensitivity to thermal load.

**Table 2:** Least squares means ± standard error of physiological and productive traits for Gyr and Girolando cows under heat stress (THI > 74) and no-stress (THI ≤ 74) conditions

Variable	No Stress		Stress	
	Gyr	Girolando	Gyr	Girolando
Rectal temperature	37.79 ± 0.042	37.78 ± 0.039	38.52 ± 0.041	38.29 ± 0.053
Respiration rate	0.63 ± 0.051	0.88 ± 0.052	1.12 ± 0.044	1.44 ± 0.048
Udder temperature	33.97 ± 0.309	34.15 ± 0.331	37.17 ± 0.305	36.92 ± 0.283
Maximum udder temperature	36.23 ± 0.218	36.84 ± 0.204	39.60 ± 0.286	39.56 ± 0.219
Minimum udder temperature	32.31 ± 0.237	32.74 ± 0.179	35.76 ± 0.172	35.75 ± 0.166
Rump temperature	31.74 ± 0.323	32.77 ± 0.294	35.59 ± 0.259	35.73 ± 0.259
Flank temperature	32.48 ± 0.235	33.13 ± 0.176	36.04 ± 0.213	36.11 ± 0.218
Neck temperature	32.19 ± 0.270	32.81 ± 0.230	35.37 ± 0.201	35.79 ± 0.203
Eye temperature	32.66 ± 0.218	33.46 ± 0.168	35.76 ± 0.188	35.94 ± 0.201
Muzzle temperature	29.36 ± 0.290	30.15 ± 0.264	33.38 ± 0.276	33.38 ± 0.227
Floor temperature	25.47 ± 0.503	25.14 ± 0.455	32.41 ± 0.448	31.79 ± 0.396
Milk production	9.38 ± 0.213	15.67 ± 0.229	9.60 ± 0.209	15.67 ± 0.223
Days in milk	161.93 ± 8.63	188.42 ± 7.30	162.02 ± 7.58	173.90 ± 6.76
Number of gestations	2.00 ± 0.037	2.19 ± 0.030	2.06 ± 0.035	2.16 ± 0.027

Gestation refers to primiparous and multiparous. Temperature variables are expressed in Celsius

### Thyroid hormone profiles

The analysis of variance revealed that the breeds had a significant effect on most thyroid hormone parameters (Table 3). Specifically, it influenced T3, T3 (nmol), and T4 free (*p* < 0.05), and showed a highly significant effect on T4 (*p* < 0.001). In contrast, the grazing system alone had no significant effect on thyroid-related variables. However, significant interactions between grazing system and breed were observed for T4 (*p* < 0.001), T4 (ng/mL) (*p* < 0.05), and T4 free (*p* < 0.001), indicating that the effect of grazing system on these hormones varied by breed. Gestation status was associated with T4 (ng/mL) only (*p* < 0.05), while time of day, days in milk, and temperature-humidity index had no significant impact on any hormone levels. Milk production was significantly associated with T4 (ng/mL) and free T4 (*p* < 0.05), suggesting a possible link between thyroid function and lactational performance. Wind speed significantly influenced T3 and T3 (nmol) concentrations (*p* < 0.001), while the ultraviolet index was associated with T4 (*p* < 0.05) and T4 free (*p* < 0.05), pointing to possible environmental modulation of thyroid hormone activity. Overall, these findings highlight the importance of breed and environmental interactions in influencing thyroid hormone dynamics, particularly T4 and its free fraction, while milk production and wind speed emerged as additional relevant factors.

**Table 3:** Summary of analysis of variance results evaluating the effects of grazing system, breeds (Gyr and Girolando), and environmental and physiological factors on Circulating hormone levels: Triiodothyronine (T3 and T3 nmol), thyroxine (T4 and T4 ng), and free thyroxine (T4 free)

Factor^1^	T3	T3 (nmol)	T4	T4 (ng)	T4 free
Grazing system	Ns	Ns	Ns	Ns	Ns
Breed	*	*	***	Ns	***
Grazing system × breed	Ns	Ns	***	*	***
Gestation	ns	ns	*	Ns	Ns
Time of day	Ns	Ns	Ns	Ns	Ns
Days in milk	Ns	Ns	Ns	Ns	Ns
Milk Production	Ns	ns	*	ns	*
THI	Ns	Ns	Ns	Ns	Ns
Wind speed	***	***	Ns	Ns	Ns
Ultraviolet index	Ns	Ns	*	Ns	*

*THI* temperature-humidity index. Gestation refers to primiparous and multiparous

F-statistics significance: *** *p* < 0.001; ** *p* < 0.01; * *p* < 0.05; *Ns* not significant

The effects of grazing system and breeds on hormone levels revealed that Girolando cows generally exhibited higher thyroid hormone concentrations than Gyr cows across both grazing systems. For instance, Girolando cows had higher T3 and T3 (nmol) levels under both shade (2.55 ng/mL and 3.92 nmol/L, respectively) and no shade (2.78 ng/mL and 4.19 nmol/L) compared to Gyr cows. T4 free levels also followed this pattern, with Girolando cows showing nearly double the concentration of Gyr cows under shade (2.38 vs. 1.36 ng/dL). Notably, while T4 concentrations were markedly higher in Girolando cows under shade (13.40 µg/dL), Gyr cows exhibited higher T4 under no shade (10.18 µg/dL vs. 10.92 µg/dL in Girolando), reflecting an interaction between genetic group and grazing system. These differences align with the significance patterns observed in the ANOVA, suggesting breed- and environment-specific endocrine responses.

### PCA of environmental and physiological traits

**Fig. 3:**
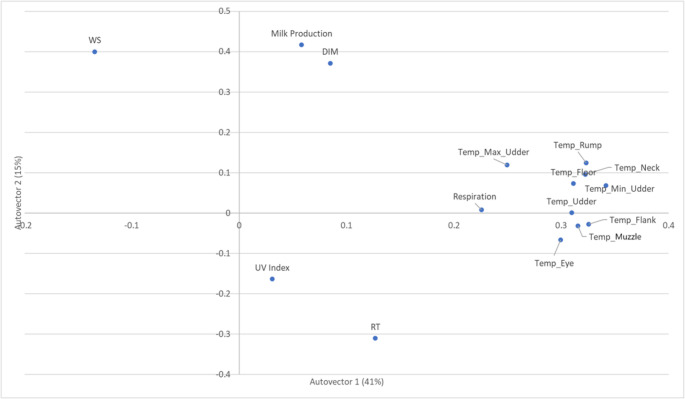
Principal component analysis biplot showing the distribution of environmental and physiological variables along the first two principal components (autovector 1 and 2). The plot illustrates each variable’s relative contribution and association, with autovector 1 and 2 explaining 41% and 15% of the variance, respectively. DIM: days in milk; WS: wind speed; UV index: ultraviolet index; Respiration: respiration rate (breaths/mim); Temp_Max_Udder: maximum udder surface temperature; Temp_Min_Udder: minimum udder surface temperature; Temp_Udder: udder surface temperature; Temp_rump: rump surface temperature; Temp_Floor: floor surface temperature; Temp_Neck: neck surface temperature; Temp_Flank: flank surface temperature; Temp_Nuzzle: Muzzle surface temperature; Temp_Eye: eye surface temperature; RT: rectal temperature. Temperature variables are expressed in celsius

The biplot of variable loadings on the first two principal components (Autovector 1 and Autovector 2), which together explain 57% of the total variance, revealed clear groupings among physiological, environmental, and production traits (Fig. 3). Surface temperature variables, including udder, rump, flank, neck, eye, and muzzle temperatures, clustered closely with respiration rate and THI, indicating a strong correlation and a shared response to thermal load. These traits loaded positively on Autovector 1, whereas wind speed was in the opposite direction, supporting its known cooling effect and inverse relationship with body temperature. Milk production and days in milk were located on the upper portion of Autovector 2, relatively distant from the stress-related traits, showing that production traits were not closely associated with immediate heat stress responses. Rectal temperature and ultraviolet index were positioned on the lower side of the plot, suggesting a potential link between internal heat load and solar radiation exposure. Overall, the analysis highlights distinct clustering of thermal stress indicators and supports the physiological relevance of surface temperature and respiration as key markers of heat stress in dairy cows.

### THI effects via broken-line regression

**Fig. 4:**
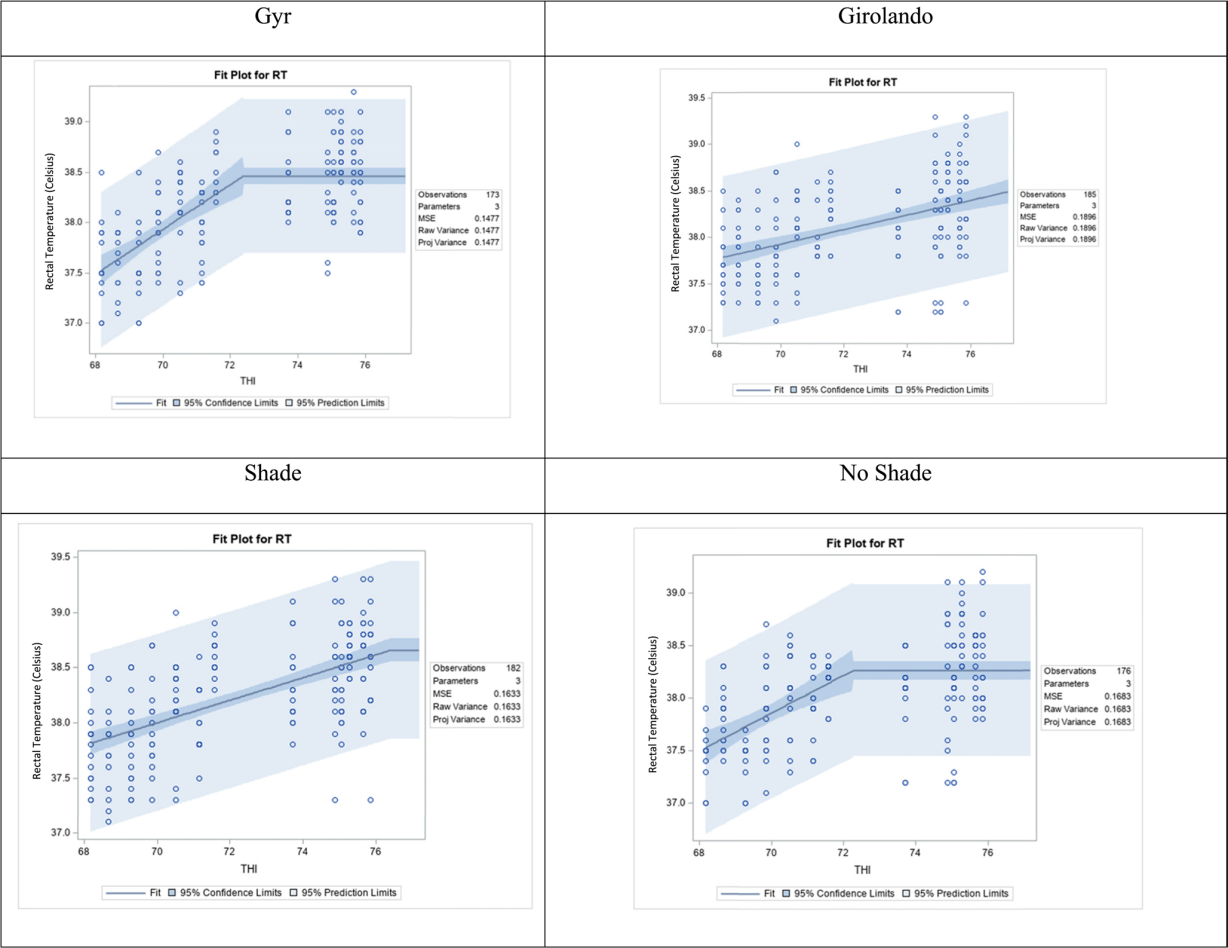
Effect of temperature-humidity index (THI) on rectal temperature (RT, celsius) in lactating cows, analyzed using segmented (broken line) regression. Each panel represents the fit plot for a different breed (Gyr and Girolando) and grazing system (shade and no shade), showing the relationship between THI and RT. The solid line represents the estimated regression, with shaded areas indicating 95% confidence and prediction intervals. Breakpoints in the response suggest changes in the physiological response of animals to increasing heat stress levels

The broken-line regression analysis (Fig. 4) revealed distinct thermoregulatory responses to increasing temperature-humidity index, based on breed and grazing system. Among breeds, Gyr cows (top left panel) showed a nonlinear response, with rectal temperature increasing with THI up to a breakpoint at THI 73.27, beyond which the slope flattened. This indicates a physiological threshold at which thermoregulatory mechanisms likely become more effective, helping to stabilise internal temperature despite further increases in environmental heat load. In contrast, Girolando cows (top right panel) exhibited a continuous linear increase in rectal temperature with no identifiable inflexion point, suggesting a reduced capacity to buffer against thermal stress and a more gradual but persistent rise in internal temperature as THI increased. These breed-specific differences highlight a more adaptive thermal response profile in Gyr cows than Girolando.

Environmental conditions further modulated these physiological responses. Under shaded conditions (bottom left panel), rectal temperature increased progressively with THI. Still, the slope remained moderate, and prediction intervals were narrower, indicating that shade contributed to more stable and uniform thermal responses among cows. In contrast, under no shade (bottom right panel), the broken-line model revealed an inflection point near THI 72, after which the increase in rectal temperature plateaued. This pattern suggests that while cows initially experienced rising rectal temperature with increasing THI, a physiological ceiling may have been reached, possibly reflecting the activation of protective mechanisms or the onset of heat stress-induced limitations. Notably, the wider prediction intervals in the no-shade condition reflect greater variability in individual responses, indicating inconsistent animal coping capacity. These findings emphasize the combined influence of genetics and microclimate on thermal regulation. Gyr cows and shaded environments contribute to a more controlled rise in rectal temperature under heat stress conditions.

**Fig. 5:**
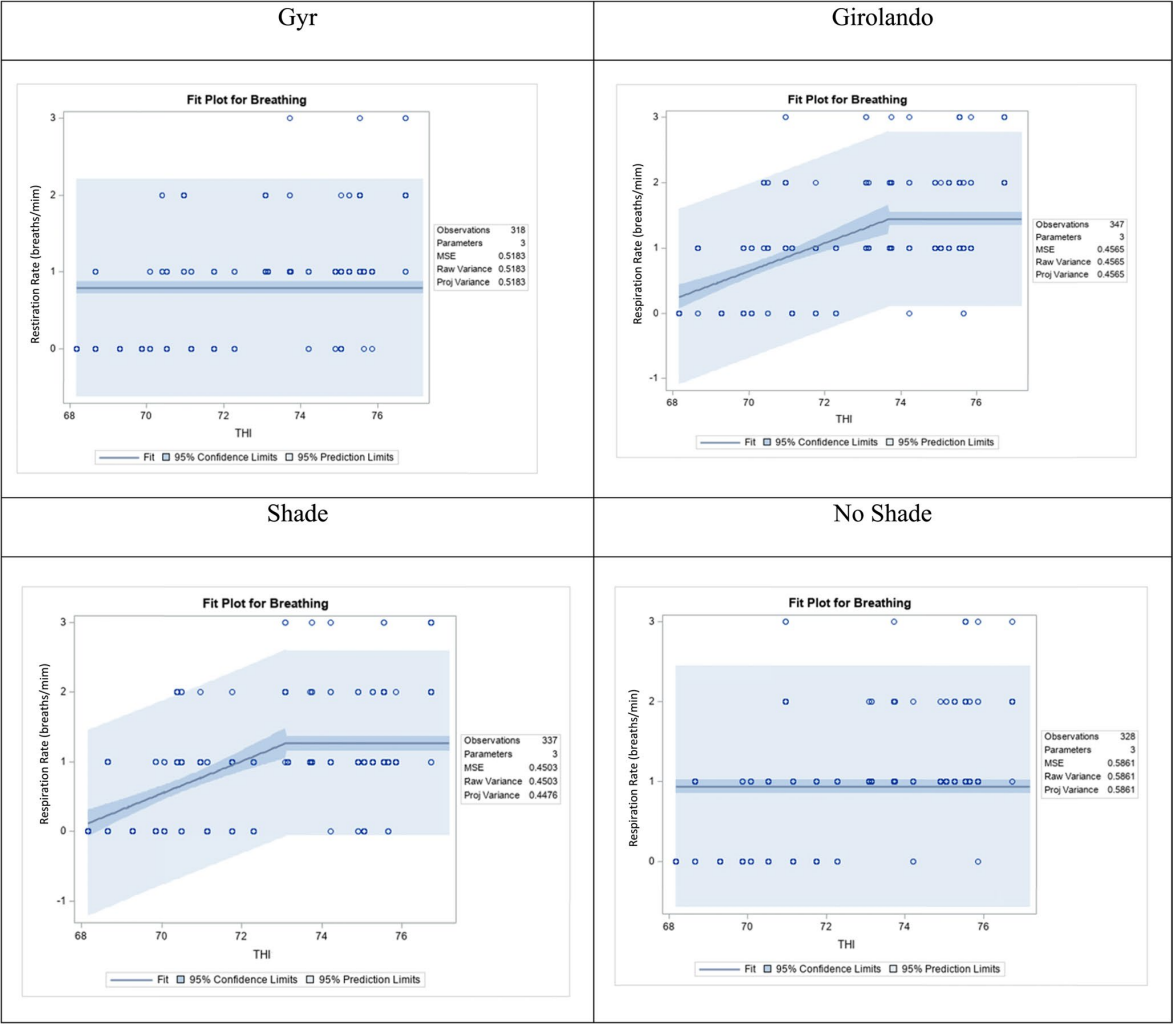
Effect of temperature-humidity index (THI) on respiration rate (breaths/min) in lactating cows, analyzed using segmented (broken line) regression. Each panel represents the fit plot for a different breed (Gyr and Girolando) and grazing system (shade and no shade), showing the relationship between THI and respiration rate. The solid line represents the estimated regression, with shaded areas indicating 95% confidence and prediction intervals. Breakpoints in the response suggest changes in the physiological response of animals to increasing heat stress levels

The results for the respiration rate as a function of THI demonstrate apparent breed differences in physiological response to heat stress. In Gyr cows (top left panel), no significant trend was observed across the THI range (Fig. 5). Respiration rates remained largely constant and were distributed across discrete classes with no evident increase as THI rose, and the regression line remained flat. This stability suggests that Gyr cows exhibit minimal change in respiration response under increasing environmental heat, supporting their reputation for greater heat tolerance and respiration efficiency. In contrast, Girolando cows (top right panel) exhibited a significant positive linear relationship between THI and respiration rate. As THI increased, breathing scores shifted toward higher values, and the regression line demonstrated an upward trend. This indicates that Girolando cows respond to thermal load with increased respiration rate, likely as a mechanism to dissipate excess body heat through evaporative cooling. The broader prediction intervals reflect greater variability in individual response, possibly due to differences in sensitivity or stage of lactation. Together, these results highlight a more robust respiration adjustment to heat in Girolando cows, in contrast to the more stable response in Gyr cows, underscoring genetic differences in coping mechanisms under heat stress conditions.

### Discriminant classification by grazing system and breed

Stepwise discriminant analysis demonstrated a clear separation between both genetic groups and grazing systems based on the variables included in the model. Regarding genetic classification, 71.15% of Gyr cows were correctly assigned to their original group, while 28.85% were misclassified as Girolando. Similarly, 71.53% of Girolando cows were correctly identified, with 28.57% misclassified as Gyr. These results show moderate discriminative power among breeds, with some traits overlapping across breeds. In contrast, classification by grazing system showed a much higher degree of accuracy. A total of 97.87% of animals under shade were correctly classified, with only 2.13% misclassified as being under no shade conditions. Likewise, 92.59% of animals under no shade were correctly identified, with a misclassification rate of 7.41% for shaded conditions. These findings suggest that the environmental condition (shade vs. no shade) had a more distinct influence on the discriminating traits than genetic group, allowing for more accurate classification of animals based on grazing environment. Variables that discriminated between breeds (Table 4) included several infrared temperatures, respiration, and rectal temperature, while for the grazing system discrimination, milk production was also a discriminant variable (Table 5).

**Table 4:** Summary of stepwise discriminant analysis identifying the most relevant variables for classifying animals according to grazing system (shade vs. no shade)

Variables^1^	Partial *R*^2^	F-value	*p*-value	Wilks’ Lambda	ASCC
Udder temperature	0.53	109.78	< 0.0001	0.47	0.53
Rectal temperature	0.14	16.39	0.00	0.41	0.59
Minimum udder temperature	0.05	5.50	0.02	0.32	0.68
Flank temperature	0.04	3.62	0.06	0.29	0.71
Respiration rate	0.03	2.42	0.12	0.28	0.72
Eye temperature	0.03	2.74	0.10	0.28	0.72

^1^Variables are listed in the order of entry into the model. Partial R² represents the individual contribution of each variable to discrimination; Wilks’ Lambda indicates overall model fit at each step; ASCC refers to the Average Squared Canonical Correlation, reflecting the cumulative discriminant power of the model. Temperature variables are expressed in Celsius

**Table 5:** Summary of Stepwise discriminant analysis identifying the most relevant variables for classifying animals according to breeds (Gyr vs. Girolando)

Variables^1^	Partial *R*^2^	F-value	*p*-value	Wilks’ Lambda	ASCC
Rectal temperature	0.06	6.47	0.01	0.94	0.06
Milk production (kg/day)	0.03	3.54	0.06	0.91	0.09
Muzzle temperature	0.03	3.09	0.08	0.88	0.12
Respiration rate	0.03	3.17	0.08	0.85	0.15
Eye temperature	0.02	2.29	0.13	0.83	0.17

^1^Variables are listed in the order of entry into the model. Partial R² represents the individual contribution of each variable to discrimination; Wilks’ Lambda indicates overall model fit at each step; ASCC refers to the Average Squared Canonical Correlation, reflecting the cumulative discriminant power of the model. Temperature variables are expressed in Celsius

### Path analysis of heat stress drivers

**Fig. 6:**
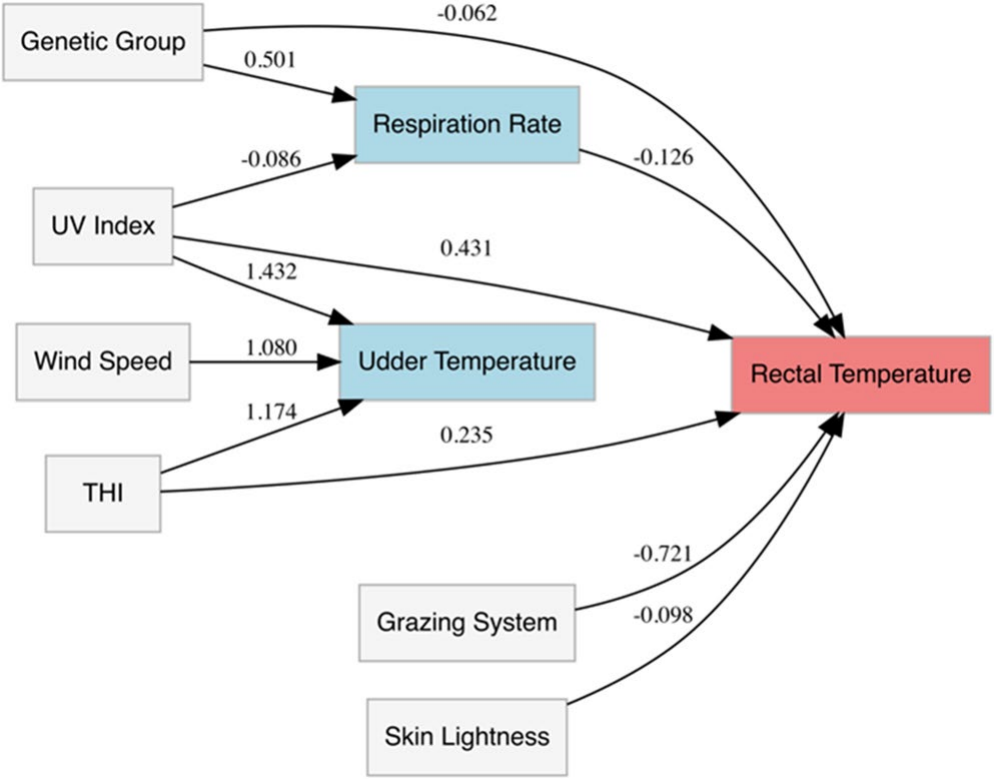
Path diagram illustrating the significant direct effects of environmental and genetic factors on physiological traits in dairy cows. Arrows represent standardized path coefficients between predictor and response variables. Node colors indicate variable type: environmental and genetic variables (e.g., THI, ultraviolet index, wind speed, grazing system, genetic group and skin lightness) are shown in light grey, intermediate physiological traits (e.g., respiration rate, udder temperature) in light blue, and the core physiological outcome (rectal temperature) in light red. All displayed paths are statistically significant (*p* < 0.05)

Path analysis revealed a network of significant direct and indirect effects influencing rectal temperature, a key indicator of heat stress in dairy cows (Fig. 6). Environmental variables including temperature-humidity index, ultraviolet radiation, wind speed, and grazing system along with intrinsic factors such as genetic group and skin lightness, exerted measurable effects on intermediate traits (respiration rate and udder temperature), influencing rectal temperature. Temperature-humidity index (THI) and ultraviolet index had strong positive direct effects on both udder temperature (β = 1.174 and β = 1.432, respectively) and rectal temperature (β = 0.235 and β = 0.431), highlighting their central role in thermal load. Wind speed also contributed positively to udder temperature (β = 1.080). Shaded grazing systems were associated with lower rectal temperature (β = − 0.721), indicating a protective thermal effect. Breed (genetic group) positively influenced respiration rate (β = 0.501), while ultraviolet index had a negative effect (β = − 0.086). Rectal temperature was further reduced by lighter skin pigmentation (β = − 0.098), breed (β = − 0.062), and increased respiration rate (β = − 0.126), suggesting these traits play adaptive roles in heat dissipation. These findings demonstrate the integrated influence of environmental conditions, genetic background, and physiological responses in determining thermoregulatory efficiency in grazing dairy cattle. Additional results of the path analysis are presented in Supplementary Table 1.

## Discussion

This study provides evidence that silvopastoral systems (i.e., shade) significantly improves thermal comfort for dairy cattle in the Cerrado biome. Across several physiological and thermographic measures, animals under eucalyptus tree shade showed consistently lower surface and rectal temperatures than those grazing in traditional grazing systems with no shade (i.e., full sun). These differences were more pronounced during periods of elevated thermal stress (THI > 74), underscoring the buffering effect of vegetative cover against environmental extremes.

The results presented here align with those of Vieira et al. (2022), who demonstrated that breeds vary in their response to heat stress; therefore, THI should be calculated independently for each breed. In general, Girolando cattle are larger than Gyr cattle (Supplementary Table 2), and have a higher milk production. Their skin colours are darker, while Lightness and tonality are lower (blacker). In the present study, the Gyr cattle generally exhibited lower respiration rates and body temperatures than Girolando cattle, particularly under heat stress conditions. This finding is consistent with previous studies reporting that animals with a higher proportion of Zebu genetics, such as the Gir breed, exhibit superior thermoregulatory adaptations to tropical environments (Cardoso et al. 2015; Carvalheira et al. 2021), including more efficient sweating mechanisms, lighter coat coloration, and shorter, sparser hair (Reis et al. 2021). However, the lack of a significant interaction between breed and production system for most traits suggests that both groups benefit from shade, although the magnitude of the response may vary.

Gyr and Girolando cattle exhibit notable differences in their resilience to heat stress, primarily due to their origins and genetic backgrounds. Gyr cattle, native to the hot and humid climate of the Gyr forest region in Gujarat, India, are well-adapted to such harsher conditions. Their large ears and loose skin facilitate thermoregulation, enabling effective heat dissipation (Pereira et al. 2014). In contrast, Girolando cattle, a hybrid breed developed in Brazil from Gyr and Holstein lineages, may show less resilience to heat stress. While they inherit some heat tolerance from their Gyr ancestry, the Holstein influence, less adapted to extreme heat, may affect their thermotolerance under high-temperature conditions (Alfonzo et al. 2016; Dalcin et al. 2016). This difference in heat tolerance can also affect milk production during peak temperatures, as Girolando cattle are primarily bred for dairy purposes (Stumpf et al. 2021). Overall, Gyr cattle generally demonstrate a higher level of heat stress resilience compared to Girolando cattle. Although no significant interaction between grazing system and breed was found for most traits, the clear physiological advantage of Gyr cattle under stress supports targeted selection. Breeding strategies that leverage heat-adapted genetic backgrounds (e.g., Gyr or composite lines with Zebu ancestry) may offer a long-term path to balancing productivity, thermal resilience, and animal welfare. It is important to note that Girolando presented higher milk production in both systems.

The lack of significant interaction for most traits does not diminish the additive benefit of shade across breeds, indicating that silvopastoral interventions can be broadly beneficial regardless of genetic background. The microclimatic conditions explain the greater presence of bovines in the silvopastoral system as they were most likely searching for the thermal comfort provided by the trees during the highest daytime temperatures (Cândido et al. 2023).

The integration of infrared thermography added a valuable dimension to the assessment, allowing for non-invasive and more efficient monitoring of specific anatomical regions (Daltro et al. 2017). The correlation between respiration rate and surface temperatures, particularly at the eye, muzzle, and udder, highlights their sensitivity as indicators of heat stress. The correlation between thermographic hotspots and respiration responses suggests the utility of thermography as a real-time diagnostic and welfare tool. Its non-invasive nature makes it scalable for precision livestock management, and its integration with artificial intelligence and remote sensing technologies could lead to advanced automated monitoring systems. Furthermore, broken line regression analyses revealed distinct THI inflection points for each breed, with Gyr cattle reaching thermal thresholds at lower THI values than Girolando, suggesting earlier physiological compensation. Broken-line regression allowed for identifying environmental thresholds, contributing to more accurate decision-making regarding heat stress mitigation strategies.

Principal component analysis (PCA) supported the observed patterns by grouping environmental and physiological variables along the first two components. Strong associations exist between surface and rectal temperatures, respiration rate, and environmental load, suggesting that these traits function as a coordinated physiological response to heat stress. This multivariate structure underscores the potential of integrative indicators for thermotolerance phenotyping. The clustering of traits suggests that animals exhibit a systemic reaction to thermal challenges, where peripheral and core temperature regulation, as well as ventilation rate, are tightly interlinked. Furthermore, separating variables along orthogonal axes may reflect distinct regulatory pathways, such as evaporative versus conductive heat dissipation, highlighting the complexity of thermoregulatory mechanisms in dairy cattle. Thus, PCA reduces dimensionality and uncovers meaningful biological structure in the response to environmental stress. These findings reinforce the value of multivariate approaches for developing composite phenotypes or selection indices that capture the multidimensional nature of thermal resilience, particularly in breeding and management strategies for climate-adapted livestock systems.

Path analysis clarified the causal relationships among traits, revealing that respiration rate and skin temperature have a direct effect on rectal temperature. In contrast, environmental variables such as temperature-humidity index, solar radiation, and wind speed influence it indirectly through these intermediate physiological responses. This finding reinforces the concept that animals do not respond to environmental stressors through isolated traits but rather through a cascade of interconnected physiological adjustments. The strong direct influence of respiration rate on core body temperature reflects the central role of respiration evaporation in thermoregulation, particularly under high heat load. Similarly, the relevance of skin temperature as a predictor of rectal temperature suggests that peripheral vasodilation and surface heat dissipation are key mechanisms of thermal balance.

These insights highlight the value of integrating causal modeling with physiological data to elucidate complex adaptive responses, particularly in field-based studies where experimental control is limited. Path analysis in this context improves our understanding of physiological integration. It identifies candidate traits, such as respiration rate and thermographic skin temperatures, that are biologically meaningful and practically measurable. As such, these traits hold promise as proxies for internal heat load, enabling the development of real-time monitoring tools and improving the feasibility of large-scale phenotyping for thermotolerance.

In tropical dairy systems, where environmental stress is a major constraint on productivity and welfare, the ability to capture internal thermal state through external indicators offers significant advantages. These findings can inform the refinement of genetic evaluation protocols by incorporating heat stress resilience as a selection objective, while also guiding management practices such as strategic shade use, water provision, and thermal stress mitigation planning. Moreover, by identifying traits that mediate the effect of environmental conditions on core physiology, this approach aligns with precision livestock farming initiatives, where predictive, individualized, and welfare-conscious decisions are increasingly necessary under climate variability.

Discriminant analysis revealed high accuracy (> 92%) in distinguishing animals based on grazing system (shade vs. no shaded), further emphasizing the systemic impact of shade on physiological responses. While variables such as rectal temperature and respiration helped distinguish breed (Gyr vs. Girolando), milk production was a more prominent discriminator in the grazing system, possibly reflecting the indirect effects of thermal stress on metabolic activity and feed intake (Chen et al. 2024). The robust classification of animals by grazing system emphasizes the systemic nature of the thermal effect of shade. This has implications not just for animal health, but for broader climate-smart agriculture strategies. Shade can mitigate productivity losses due to thermal stress, support biodiversity, and contribute to carbon sequestration, making silvopastoral systems attractive for policy incentives or environmental certification programs. Beyond animal welfare, such systems also provide ecological co-benefits, contributing to more resilient, multifunctional production landscapes.

Thyroid hormone analysis showed a significant interaction between genetic group and system for T4 but not T3, indicating a nuanced endocrine response to thermal load. This hormonal modulation may contribute to the ability of the animals to maintain homeostasis under different environmental conditions, although further investigation into these pathways is warranted (Anjali et al. 2023). The elevation of T4 in Girolando under shade versus sun implies endocrine-driven modulation of metabolism. While this may help maintain productivity, it could also signal higher metabolic costs associated with thermal compensation, which might influence reproductive health and longevity. The endocrine responses merit further study as potential biomarkers for resilience. These endocrine responses warrant further investigation as potential biomarkers of resilience.

These findings underscore the importance of incorporating tree shade into pasture-based systems, particularly in climates prone to thermal extremes. The clear benefits observed in both breeds suggest that silvopastoral systems represent a viable strategy to enhance animal welfare and productivity in tropical dairy production systems. Future studies should investigate the long-term effects on milk yield, fertility, and immune function, and quantify the economic trade-offs associated with tree planting, maintenance, and land use. Behavioural observations (e.g., time spent in shade, feeding patterns) and expanded sampling across seasons would provide a more holistic view of system performance.

## Conclusion

The results of this study demonstrate that silvopastoral systems incorporating *Eucalyptus urograndis* can significantly improve thermal comfort in dairy cattle raised under tropical conditions. Shade reduced surface and rectal temperatures and panting scores in both Gyr and Girolando breeds, particularly during periods of high thermal stress. While Gyr cattle showed greater natural tolerance to heat, the benefits of shade were evident for both genetic groups, indicating that silvopastoral strategies enhance animal welfare regardless of breed. Discriminant and regression analyses further highlighted the systemic physiological and endocrine differences driven by shade and genetic background. These findings support the broader adoption of tree-based pasture systems as an effective adaptation to mitigate climate-related heat stress in tropical livestock production. Future research should investigate the long-term effects on productivity, health, and reproduction, and assess the economic viability of implementing silvopastoral systems on a large scale.

## Supplementary Information

Below is the link to the electronic supplementary material.

ESM 1 (DOCX 21.0 KB)

## Data Availability

Data presented in this study are available on reasonable request from the corresponding author.
