# Supplementary material for: Shade matters: heat stress alleviation in Gyr and Girolando cows through silvopastoral management in tropical conditions
Source: Int J Biometeorol. 2026 Jan 14;70(1):27. doi: 10.1007/s00484-025-03063-7 (PMC12804218; doi:10.1007/s00484-025-03063-7)
Supplement: Supplementary file 1 — (DOCX 21.0 KB) [file 484_2025_3063_MOESM1_ESM.docx]

Supplementary Table 1 Standardized path coefficients from the path analysis model assessing the direct effects of environmental and genetic factors on physiological traits in dairy cows

|  | **Pathway** |  | **Estimate** | **p-value** |
| --- | --- | --- | --- | --- |
| Breed | ===> | Respiration Rate | 0.501 | <.0001 |
| Ultraviolet index | ===> | Respiration Rate | -0.086 | <.0001 |
| THI | ===> | Udder Temperature | 1.174 | <.0001 |
| Ultraviolet index | ===> | Udder Temperature | 1.432 | 0.024 |
| Wind speed | ===> | Udder Temperature | 1.080 | 0.010 |
| THI | ===> | Rectal Temperature | 0.235 | <.0001 |
| Ultraviolet index | ===> | Rectal Temperature | 0.431 | <.0001 |
| Grazing system | ===> | Rectal Temperature | -0.721 | <.0001 |
| Breed | ===> | Rectal Temperature | -0.062 | <.0001 |
| Lightness skin | ===> | Rectal Temperature | -0.098 | <.0001 |
| Respiration rate | ===> | Rectal Temperature | -0.126 | <.0001 |

THI – temperature, humidity index. Only statistically significant paths (p < 0.05) are shown

Supplementary Table 2 Means for breed variables

| **Variable** | **Breed** | |
| --- | --- | --- |
|  | **Gyr** | **Girolando** |
| Score | 3.38 | 3.11 |
| Shoulder length (m) | 0.64 | 0.66 |
| Back height (m) | 0.98 | 1.02 |
| Flank height (m) | 0.88 | 1.07 |
| Leg height (m) | 1.18 | 1.04 |
| Hair thickness | 0.92 | 0.95 |
| Skin thickness | 0.62 | 0.63 |
| Body length (m) | 1.43 | 1.63 |
| Hip height (m) | 1.29 | 1.42 |
| Canon bone circumference (cm) | 21.15 | 21.74 |
| Back circumference (m) | 1.77 | 2.06 |
| Lightness skin | 24.65 | 23.84 |
| Colour skin | 9.27 | 5.52 |
| Tonality skin | 50.23 | 55.64 |
| Lightness scapula | 40.95 | 21.78 |
| Colour scapula | 17.96 | 8.53 |
| Tonality scapula | 68.42 | 72.38 |
| Lightness 13th rib | 38.27 | 23.12 |
| Colour 13th rib | 17.48 | 7.98 |
| Tonality 13th rib | 68.30 | 73.14 |
| Lightness rump | 37.77 | 20.99 |
| Colour rump | 19.80 | 8.48 |
| Tonality rump | 67.21 | 72.43 |
